# Suicide attempt in a rural area of Vietnam: Incidence, methods used and access to mental health care

**DOI:** 10.1186/1752-4458-4-3

**Published:** 2010-02-17

**Authors:** Tuan V Nguyen, Christina Dalman, Thien C Le, Thiem V Nguyen, Nghi V Tran, Peter Allebeck

**Affiliations:** 1 Department of Public Health Sciences, Karolinska Institute, Stockholm, Sweden; 2 Department of Psychiatry, Hanoi Medical University, Hanoi, Vietnam; 3 National Institute of Mental Health, Hanoi, Vietnam

## Abstract

**Objectives:**

The study aims to determine the incidence of suicide attempt, describe the methods used, and assess use of health care services including mental health care after suicide attempt in a rural area of Vietnam.

**Methods:**

All suicide attempters (104) during 2003-2007 were listed, diagnosed and re-evaluated by trained physicians according to the research criteria of the WHO Multicentre Study of Attempted Suicide. All attempters were interviewed by trained medical staff to investigate methods used, socio-demographic characteristics and use of health services.

**Results:**

The yearly incidence was 10.2 per 100000 person-years, 10.6 per 100000 in males and 9.8 per 100000 in females. 99% of cases committed suicide attempt by poisoning, 62.6% by pesticides and 36.3% by pharmaceutical drugs. 34.3% reported having been in contact with somatic care and 13.2% had received mental health care. Among those who reported some treatment received, 47.5% had been in contact with official health care services, 8.1% had pharmacy keepers' consultation or were treated by traditional healers and 4% reported self treatment.

**Conclusion:**

The incidence of suicide attempt was lower in this population compared to other settings. While the majority of attempters use pesticides, many had used psychotropic drugs. Contact with mental health services following the attempt was very limited in this setting. Suicide prevention for this high risk group should focus on reducing access to pesticides and psychotropic drugs. Mental health services should be made more accessible in rural areas.

## Introduction

Suicide attempt is both one of the strongest risk factors for completed suicide and an important indicator of extreme emotional distress [1]. There are no figures on the exact number of suicide attempts in the world as most countries lack monitoring systems. Studies on suicide attempt are most often conducted in hospital settings or in epidemiological catchments areas. The estimated average annual rates of suicide attempt in recent decades range from 2.6 to 1,100 per 100,000 person-years [2]. There are differences in suicide attempt rates between countries; for example, the rate has been estimated to 357-534 per 100,000 in Canada, 900-1100 per 100,000 in Finland, 49-81 per 100,000 in India, and 41-96 per 100,000 in Singapore [2-4]. However, in many countries, data is lacking, and this is especially the case for South East Asia. Especially in these countries with strong social and economic transition, further studies are needed to follow trends and patterns of suicidal behaviour.

Self-poisoning is the predominant method of suicide attempts in most low and middle income countries, by far exceeding methods such as shooting, cutting and hanging [5]. Most cases of self-poisoning involve the ingestion of pesticides or pharmaceutical drugs [5,6]. The majority of suicide attempters in urban areas use psychotropic drugs such as tranquillisers and anti-psychotics [7], whereas in rural areas, pesticides or other chemicals used in agriculture dominate [6,8]. In a previous article [7] we showed that methods used vary between urban and rural areas of Vietnam. It is of interest to find out whether there is a shift in methods used also in rural areas, where some groups move from mainly farming to other occupations and salary work.

Basically, the same risk factors have been identified for completed suicides as for suicide attempts, such as availability of methods used (e.g. drugs, weapons), mental disorders and socio-demographic characteristics, e.g. living alone, unemployment and lack of social support [9-11]. Since risk factors often remain unchanged in persons who have attempted suicide, there is a considerable risk of recurrence. Appropriate care and follow up of persons who have attempted suicide is thus important, especially since it has been shown that around 90% of attempters have a psychiatric diagnosis [1] and more than 50% have physical sequelae which require medical attention or surgery [5,12,13]. Again, while primary and secondary care services are well developed in many low and middle income countries in South East Asia, the status and capacity of mental health services is less developed, and need to be improved.

The aims of the present study were 1) to determine the incidence of suicide attempt in a rural area of Vietnam, 2) to describe the methods used, and 3) to assess the socio-demographic conditions and use of health care services, in particular mental health treatment after the index attempt.

## Methods

### Setting

The study was conducted in a rural area of Gia Luong in Bac Ninh province, located in the Red River Delta of the northern part of Vietnam. The area covers a land area of 208.7 km^2^. The mean population during the study period was 204,000 persons, making the population density very high for a rural area, over 1,200 persons per square kilometer. The majority of residents are farmers. Others are civil servants, manual workers, and small traders. The main economic activity in the area is farming. During the last years, a growing number of persons have left after harvest time to work as temporary workers in the city or in other provinces. The major ethnic group is Kinh, which is the biggest group in Vietnam [14].

The health care system in this area is organized according to the national system of rural health services Vietnam. The public health care services consist of two district hospitals, with approximately 120 beds in each, and 29 community health stations. District hospitals are mainly responsible in curative care, while community health stations are responsible for primary health care including essential curative care and preventive care. There are small pharmacies available in each community health station and only a few private pharmacies are located in this area.

### Data collection

We identified persons who had been admitted to the district hospitals, as well as all health care stations, during 2003-2007 in Gia Luong area after having committed either a definite suicide attempt or an uncertain suicide attempt according to ICD-10. In addition, cases that were treated at home by medical staff from the community health center after suicide attempt were also included. For persons who had committed more than one suicide attempt, the first attempt was defined as index attempt. Each case of suicide attempt was diagnosed and re-evaluated by trained physicians according to the research criteria of the WHO Multicentre Study of Attempted Suicide [15].

Data was collected retrospectively in the beginning of 2008 based on records kept at the health services and interviews with medical staff.

All persons who had committed suicide attempt were interviewed face-to-face in their home by trained medical staff working at the community health stations. We used the instrument developed by the WHO Multicentre Study [15] to record data on age, gender, marital status, types of living, occupation, date of the event, perceived need of support and use of health care services after the index attempt.

The study was approved by the Institutional Review Board of Hanoi Medical University

### Statistical analyses

Data analyses were performed using SPSS for Window Version 10.0 (SPSS, Chicago. IL, USA). Statistical significance for differences between groups was assessed by chi-square test. The annual incidence rates were based on a mean population of 204,000 inhabitants. Analyses are based on the index attempt during the studying period. Due to difficulties in finding an appropriate control group, we report results comparing with sociodemographic data from a random sample in a similar rural area in Vietnam [16].

## Results

### Incidence and methods used of suicide attempt

We identified altogether 104 individuals, 54 males and 50 females, who committed suicide attempt during the study period. Of these, 90 persons were identified through hospital records, and 14 identified by medical staff after treatment at home. Including repeated attempts, totally 116 suicide attempts were performed during the study period. The yearly incidence of first attempt was 10.2 per 100,000 person-years, 10.6 per 100,000 in males and 9.8 per 100,000 in females.

Poisoning was the method used in 99% of the cases (table 1). The most common method of poisoning was by pesticides (62.6%), followed by pharmaceutical drugs (36.3%).

**Table 1:** Methods used at the index attempt

	Index attempt	
	
	N	%
**X60: **Non-opiate analgesics, antipyretics and ant rheumatics	3	3.0
**X61: **Antiepileptic, sedative-hypnotic, anti-parkinsonism and psychotropic drugs	33	33.3
**X68: **Pesticides	62	62.6
**X78: **Using blunt objects	1	1.0

**Total**	**99**	**100.0**

### Socio-demographic characteristics

The large majority of suicide attempters were living together with their family members or others (83.8%). Only 3% were living alone.

Being single or widowed/divorced/separated was reported by 34.3%, which is not significantly different from the general rural population in Vietnam (28.8%). Around 14% of our subjects were unemployed, which is somewhat higher than in the general rural population in Vietnam (7.3%).

### Need and support from the family and the community

Emotional support was reported needed to a larger extent than practical support from both the family and the community (table 2). The individuals felt that they received more support, both practical (72%) and emotional (85%), from the family compared to the community (21% and 46%, respectively).

**Table 2:** Needs and supports from family, friends and community

	Family			Friends			Community		
	
	No, not at all N (%)	To some extent N (%)	Yes, very much N (%)	No, not at all N (%)	To some extent N (%)	Yes, very much N (%)	No, not at all N (%)	To some extent N (%)	Yes, very much N (%)
**Practical support**									
Do you feel that you need practical support?	36 (36.4)	36 (36.4)	27 (27.3)	60 (60.6)	34 (34.3)	5 (5.1)	58 (58.6)	35 (35.4)	6 (6.1)
Do you feel that you get the practical support you need?	28 (28.3)	44 (44.4)	27 (27.3)	68 (68.7)	29 (29.3)	2 (2.0)	78 (78.8)	18 (18.2)	3 (3.0)

**Emotional support**									
Do you feel that you need emotional support from?	17 (17.2)	35 (35.4)	47 (47.5)	34 (34.3)	45 (45.5)	20 (20.2)	41 (41.4)	39 (39.4)	19 (19.20
Do you feel that you get the emotional support you need?	15 (15.2)	42 (42.4)	42 (42.4)	36 (36.4)	54 (54.5)	9 (9.1)	54 (54.5)	39 (39.4)	6 (6.1)

### Use of health care services

A large proportion (44.4%) refused to be referred to (table 3). Only about one third (35.4%) accepted referral to professional care, and 22.2% were not sure if they accepted or had no referral. Altogether 35 persons received care from the official health care services such as hospitals, community health stations and private health care providers. A minority sought help from traditional healers (4 persons) or pharmacies/self-treatment (8 persons). Around one third (34.3%) reported having been in contact with somatic care and 13.2% had received mental health care.

**Table 3:** Uses of health care services after the index attempt, by sex

		Male N	Female N	Total N (%)
Referral to professional care	Acceptance	19	16	35 (35.4)
	No referral to professional care and/or not sure of acceptance for referral to professional care	12	10	22 (22.2)
	Refusals	21	21	44 (44.4)
	**Total**	**52**	**47**	**99 (100.0)**

Types of health care	Hospitals/Community health station/Private health care providers	20	15	35 (35.4)
	Traditional healers	2	2	4 (4.0)
	Pharmacy keepers/Self-treatments	4	4	8 (8.1)
	**No health care**	**26**	**26**	**52 (52.5)**
	**Total**	**52**	**47**	**99 (100.0)**
Types of treatment	In-patient somatic treatment	9	3	12 (12.1)
	Out-patient somatic treatment	8	14	22 (22.2)
	In-patient psychiatric treatment	5	1	6 (6.1)
	Out-patient psychiatric treatment	4	3	7 (7.1)
	**No treatment**	**26**	**26**	**52 (52.5)**
	**Total**	**52**	**47**	**99 (100.0)**

## Discussion

The incidence rate of suicide attempt was 10.2 per 100,000 person-years in our study. Our incidence is lower compared to countries such as Canada (357-534 per 100,000), Norway (90-149 per 100,000), and Singapore (41-96 per 100,000), but higher compared to Nigeria (2.6 per 100,000) [2,4]. Lack of previous community-based research precludes us from concluding whether the incidence rate of suicide attempt in rural Vietnam have increased or decreased in response to the country's rapid socioeconomic change.

There was no major gender difference in rates of suicide attempt in our study (male/female ratio 1.1:1). Studies from Nigeria and Finland showed higher ratios, 1.4:1 and 1.3:1, respectively [17]. Lower ratios (more suicide attempts in females) have been reported from England (1:1.2), France (1:1.9), and Singapore (1:2.3) [2,15].

The most common method used was poisoning. Pesticides are still the most common method for intoxication in suicide attempts in rural Vietnam. This is in agreement with what has been reported from other developing countries, such as China, Malaysia, and Uganda [18]. The existence of toxic pesticides in most farmers' homes without security arrangements makes pesticides easily accessible. Pesticides are also cheaper and more easily available compared to pharmaceutical drugs and other chemicals used for intoxication [8]. According to a WHO report, the use of pesticides is now the most common method of suicide worldwide [19]. The most likely explanation for the high numbers of pesticides suicides in developing countries is the high lethality associated with pesticides ingestion compared to the relatively low lethality of many of the substances commonly taken in acts of self-poisoning in the West [20]. The urbanization seems to be associated with the transition of the methods of poisoning used from pesticides to pharmaceutical drugs, which has recently been reported from Sri Lanka [21]. The fact that a considerable proportion of suicide attempt was by pharmaceutical drugs may indicate a higher access to more expensive and less lethal means of suicide attempt also in rural Vietnam, may be as part of the socio-economic transition [7,8].

Socio-demographic characteristics were not very different from general population. There were only 3% living alone and around 14% unemployed in our study. The rate of unemployment among our cases was lower compared to findings from developed countries, although similar to what has been found in China [5,22]. It should be noted that unemployment may not have the same meaning in Vietnam as in developed countries, since many people can find temporary jobs in farming or simple manual work. Thus stigma associated with unemployment may be different in societies less dependent on salaried employment.

Need of support from the family was reported to be higher than support from the community. This is contrary to findings from high income countries, in which the need of support from the community has been showed to be more important than from the family [10]. This could be explained by the differences in supply of services between low, middle income and rich countries. Emotional support was more needed than practical support, from the family as well as the community. This suggests that the problems perceived by persons who have attempted suicide are less medical or psychiatric, but more of psychosocial nature [23].

Ambivalence to treatment and early dropout were well-known problems among suicide attempters [6,24]. However, the proportion who accepted referral to professional care in this study was even lower than in other studies [25]. Unfortunately, we did not ask specifically about reasons for this. One reason could be that people who attempted suicide in Vietnam did not consider their problems as mental health or medical and doubt the appropriateness of contact with mental health services [26]. Another hypothesis is that the stigmatization concerning suicide attempt in low and middle income countries such as Vietnam is larger compared to western countries. Integrating mental health care into primary health care could be one way to improve the care of people who have attempted suicide or who have other mental health problems [27]. Also, physicians and staff of community health centers as well as district hospitals should be more aware of mental health problems in the community. Policy and training should aim to reduce stigma associated with mental illness as well as general psychological distress.

### Limitations

The present study provides new findings on the incidence, methods used and use of health care services of suicide attempters after the index attempt in rural Vietnam. Some limitations in our study need to be acknowledged: First, Vietnam consists of rural and urban areas with important sociocultural differences, and thus differences in patterns of suicidal behaviour [7]. Consequently, our findings may at best be generalized to the rural population. Second, socio-demographic characteristics, supports and use of health care services were assessed by retrospective self-report without independent validation. Although systematic reviews have shown that adults can recall past experiences with sufficient accuracy [28], recall bias arising for various reasons can not be excluded. Third, suicide attempt is still stigmatized in Vietnam. Thus, there might be cases that were not in contact with health services and not reported. This is however not unique to Vietnam but a problem common to all studies on suicide attempts. Finally, we used the WHO Multicentre Study instrument that has been translated and previously used in Vietnam [16], but not rigorously validated.

## Conclusion

The incidence of suicide attempt was lower in this population compared to other settings. The majority of attempters use pesticides, which is an important target for preventive strategies. Strategies of suicide prevention should comprise limiting the access to pesticides by, for example, raising public awareness of the need to keep these potential poisons safely locked in and available only to authorized persons. The increasing use of psychotropic drugs indicates a need for stronger regulation of the accessibility to these drugs. Policy makers should be aware of the risk of "swapping" to other methods, which is why limiting access to means of suicide only can be a part of any suicide prevention program.

The proportion of individuals who had contact with mental health services following the attempt was very limited in this setting, common to many low and middle income countries. Better access to mental health care should be prioritized for this group, possibly by integration of mental health care into primary health care. A national policy highlighting training and awareness of mental health problems in the community should be advocated.

## Competing interests

The authors declare that they have no competing interests.

## Authors' contributions

All authors contributed to study design and planning of data collection. TVN and TCL performed the data collection. TVN, PA and CD had main responsibility for analyzing data and writing the report. TVN was principal author of the paper. All authors have approved the final manuscript.
